# Acute differences in field-based physical performance between Ramadan-type fasting and non-fasting conditions in 10–11-year-old youth soccer players

**DOI:** 10.3389/fspor.2026.1943164

**Published:** 2026-10-01

**Authors:** Yılmaz Yüksel, Mehmet Emir Mentaş, Furkan Karahan, Tuba Kızılet Topateş, Salih Çabuk, Kubilay Topçuoğlu, Serhat Özbay, Süleyman Ulupınar

**Affiliations:** 1 Department of Coaching Education, Faculty of Sports Sciences, Istanbul Topkapi University, Istanbul, Türkiye; 2 Institute of Health Sciences, Erzurum Technical University, Erzurum, Türkiye; 3 Department of Coaching Education, Faculty of Sports Sciences, Marmara University, Istanbul, Türkiye; 4 Department of Coaching Education, Faculty of Sports Sciences, Erzurum Technical University, Erzurum, Türkiye; 5 Department of Physical Education and Sports, Faculty of Sports Sciences, Erzurum Technical University, Erzurum, Türkiye

**Keywords:** intermittent endurance, perceived exertion, Ramadan fasting, sleep duration, youth soccer, Yo-Yo test

## Abstract

**Introduction:**

Ramadan-type fasting involves prolonged abstinence from food and fluids and may influence physical performance, fatigue, hydration-related responses, and sleep. However, evidence regarding its acute effects on field-based performance in preadolescent soccer players remains limited. This study aimed to compare physical performance, physiological and perceptual responses, sleep duration, and body mass between fed and Ramadan-type fasted conditions in 10–11-year-old male youth soccer players.

**Methods:**

Fifteen male youth soccer players completed a within-subject repeated-measures design under two nutritional conditions: fed and Ramadan-type fasted. In the Ramadan-type fasted condition, participants abstained from food and fluid intake for approximately 14 h before testing, whereas in the fed condition testing was performed approximately 3.5–4 h after a standardized meal. Field-based performance was assessed using countermovement jump, 30-m sprint, Illinois agility test, and the Yo-Yo Intermittent Recovery Children's Test. Resting heart rate, pre-test subjective fatigue, post-Yo-Yo perceived exertion, sleep duration, and body mass were also recorded.

**Results:**

Countermovement jump height, 30-m sprint time, and Illinois agility test time did not differ significantly between conditions. In contrast, Yo-Yo Intermittent Recovery Children's Test distance was significantly lower under the fasted condition compared with the fed condition. Resting heart rate, pre-test subjective fatigue, and post-Yo-Yo perceived exertion were significantly higher in the fasted condition, while sleep duration and body mass were significantly lower.

**Discussion:**

These findings indicate that the Ramadan-type fasted condition was associated with lower intermittent endurance performance and greater perceptual strain, whereas no statistically significant differences were detected in isolated short-duration performance tasks in child soccer players. Coaches and practitioners should carefully monitor fatigue, perceived exertion, sleep, and recovery when planning intermittent endurance-oriented activities for children who voluntarily fast with parental approval.

## 1 Introduction

Ramadan fasting is characterized by abstinence from food and fluids from the pre-dawn meal until sunset and represents a distinctive model of intermittent fasting that may influence daily nutritional intake, hydration opportunities, sleep patterns, and physical activity routines (1–3). In athletic populations, these changes may have practical relevance because training and performance outcomes are closely linked to energy availability, fluid balance, recovery, and perceived fatigue (4, 5). This issue may be particularly important in youth athletes, whose physiological responses occur within the context of ongoing growth, maturation, and sport-specific development (6, 7). Therefore, examining Ramadan-type fasting in child soccer players may provide useful insights into how short-term food and fluid restriction is associated with field-based physical performance under ecologically relevant conditions.

The potential influence of fasting on physical performance is likely to vary according to the physiological and neuromuscular demands of the task (1, 2, 8). Short-duration explosive actions, such as jumping and linear sprinting, rely largely on neuromuscular coordination, rapid force production, and immediate energy supply and may therefore be relatively less affected by acute fasting conditions (4, 5). In contrast, intermittent endurance performance may be more sensitive to food and fluid restriction because it requires repeated high-intensity running, cardiovascular regulation, thermoregulatory control, recovery between efforts, and sustained motivation (8, 9). Accordingly, Ramadan-type fasting may not affect all performance outcomes uniformly; rather, its effects may be more evident in tasks with greater cumulative physiological and perceptual demands. During prolonged high-intensity intermittent exercise, reduced carbohydrate availability, declining blood glucose, and progressive muscle-glycogen utilization may compromise repeated-effort capacity and recovery between bouts. In children, maturation-related differences in substrate use and glycolytic capacity may modify these responses (10, 11). However, blood glucose and muscle glycogen were not measured in the present study.

Soccer performance in youth players is multidimensional and depends on the interaction of explosive power, linear sprint ability, change-of-direction performance, and intermittent endurance capacity (12, 13). Field-based tests are commonly used to evaluate these components because they are practical, sport-relevant, and applicable in training environments (14, 15). Countermovement jump performance reflects lower-body explosive capacity, whereas 30-m sprint performance reflects linear acceleration and sprint ability (16, 17). Change-of-direction tests, such as the Illinois agility test, assess the ability to decelerate, reaccelerate, and control body position during rapid directional changes (18, 19). In addition, the Yo-Yo Intermittent Recovery Children's Test provides a practical assessment of intermittent endurance capacity, particularly relevant to soccer, which requires repeated bouts of high-intensity running interspersed with short recovery periods (20–22).

Although the effects of Ramadan fasting on athletic performance have been examined in different age groups and sport contexts (4, 8, 23), evidence in 10–11-year-old youth soccer players remains limited. Moreover, many previous studies conducted during Ramadan include concurrent changes in sleep timing, daily routines, training schedules, and environmental conditions, making it difficult to isolate the acute association between fasting status and field-based performance (2, 3). Ramadan-type fasting is not limited to the month of Ramadan; it may also be voluntarily practiced for a few days on specific religious occasions outside Ramadan. These short-term fasting periods may provide a suitable context for examining the acute effects of food and fluid abstinence, as they are less likely to involve the broader lifestyle alterations or potential chronic adaptations associated with the full month of Ramadan. Therefore, a Ramadan-type fasting model performed outside the calendar month of Ramadan may provide a more focused approach for comparing fasted and non-fasted conditions, provided that fasting is voluntary and not imposed by the researchers. Accordingly, this study aimed to compare field-based physical performance, resting heart rate, subjective fatigue, perceived exertion, sleep duration, and body mass between Ramadan-type fasted and fed conditions in 10–11-year-old male youth soccer players.

## 2 Material and methods

### 2.1 Study design

This study used a randomized and approximately balanced within-subject repeated-measures design to compare field-based physical performance between fed and Ramadan-type fasted conditions in 10–11-year-old male soccer players. Yo-Yo Intermittent Recovery Children's Test distance was designated *a priori* as the primary outcome in the ethics-approved study protocol; all remaining variables were secondary outcomes. Each participant completed four experimental testing sessions: one short-duration performance session and one intermittent-endurance session under each nutritional condition. The order of the nutritional conditions was randomized and approximately balanced, with eight participants completing the fed condition first and seven completing the Ramadan-type fasted condition first. Consecutive experimental sessions were separated by 72–96 h. Within each participant, the order of the short-duration and intermittent-endurance sessions was maintained across conditions. All experimental sessions were conducted at approximately 17:00 to minimize variation related to time of day. Participants completed a familiarization session 5–7 days before the first experimental session.

### 2.2 Participants

The original *a priori* sample-size calculation was conducted in G*Power software version 3.1.9.4 using a one-tailed paired-samples t-test, an effect size dz of 0.60, an alpha level of 0.05, and statistical power of 0.80, indicating a required sample of 19 participants. Following adoption of the more conservative two-tailed analytical framework in the revised manuscript, the same assumptions yielded a required sample of 24 participants. Although 20 participants were initially enrolled, only 15 completed all testing procedures in both conditions and were included in the final analysis. Thus, the final sample did not reach either target, limiting statistical power and precision, particularly for small-to-moderate effects. Of the five non-completers, two did not wish to complete the testing procedures, one decided not to undertake the fasted condition, and two voluntarily withdrew from the study.

Participants were eligible for inclusion if they were male soccer players aged 10–11 years, actively participating in regular soccer training, and able to complete both fed and Ramadan-type fasted testing conditions. Children were excluded if they had any known cardiovascular, metabolic, neurological, or musculoskeletal condition that could affect exercise performance or increase the risk associated with testing. Participants who reported acute illness, injury, or any condition that prevented maximal physical effort during the testing period were excluded from analysis. The descriptive characteristics of the participants were as follows: age, 10.40 ± 0.51 years; height, 143.4 ± 4.7 cm; body mass, 36.9 ± 3.7 kg; body mass index, 18.00 ± 2.2 kg/m^2^; and body fat percentage, 9.2 ± 2.1%. These anthropometric measurements were obtained during the familiarization visit and represent the baseline characteristics of the 15 participants who completed both experimental conditions and were included in the final analysis. All participants had at least two years of organized soccer experience and regularly completed two to three supervised soccer-training sessions per week, corresponding to approximately 180–270 min of structured training per week. Participants also played age-group matches during the competitive season. Experimental testing was scheduled outside the match calendar. Participants were familiarized with all field-based testing procedures before data collection.

Only children who voluntarily practiced Ramadan-type fasting with parental approval were included in the fasted condition. No participant was instructed or encouraged by the researchers to fast for the study. Final participation required written parental consent and verbal/written assent from the child.

### 2.3 Ethical considerations

The study protocol was reviewed and approved by the Scientific Research and Publication Ethics Committee of Erzurum Technical University before data collection (Meeting date: 30 April 2026; Meeting no: 5; Decision no: 2026-ETÜ-0131). All procedures were conducted in accordance with the principles of the Declaration of Helsinki. Because the participants were children, written informed consent was obtained from their parents or legal guardians, and each child provided assent before participation. Participation was entirely voluntary, and the children were informed that they could withdraw from the study at any time without providing a reason. The researchers did not instruct, encourage, or require any participant to fast. The Ramadan-type fasting condition included only children who had voluntarily chosen to fast with parental approval. Therefore, the research team did not impose fasting as an experimental intervention. Before each testing session, participants underwent a brief health screening to confirm their ability to perform the planned field-based tests. Testing was not performed if a participant reported illness, injury, dizziness, unusual fatigue, or any condition that could increase the risk of exercise. During testing, the protocol was stopped immediately if a participant reported dizziness, nausea, excessive fatigue, discomfort, or unwillingness to continue. A responsible adult and research staff were present throughout all testing sessions to monitor participant safety.

### 2.4 Experimental conditions

Participants completed the same testing procedures under two nutritional conditions: fed and Ramadan-type fasted. In the Ramadan-type fasted condition, participants consumed a standardized pre-dawn meal completed between 02:30 and 03:00. The meal provided approximately 2.0 g/kg carbohydrate, 0.3 g/kg protein, and 0.25 g/kg fat, together with approximately 7 mL/kg of water. Its composition was matched as closely as possible to the standardized meal used in the fed condition. Each participant completed food and water intake near 03:00 and subsequently abstained completely from both until testing at approximately 17:00. The exact intervals since the last food and fluid intake were recorded separately because individual completion times differed slightly. Compliance with the fasting period was confirmed verbally with both the child and parent before testing. Although data collection occurred outside the calendar month of Ramadan, this condition reproduced the typical food- and fluid-abstinence pattern of Ramadan fasting.

In the fed condition, participants consumed a standardized pre-exercise meal completed approximately 3.5–4 h before testing. The meal provided approximately 2.0 g/kg carbohydrate, 0.3 g/kg protein, and 0.25 g/kg fat, corresponding to approximately 420–450 kcal for a participant of average body mass. Participants also consumed approximately 7 mL/kg of water with the meal. The meal was predominantly carbohydrate-based, moderate in protein, and relatively low in fat and dietary fiber to facilitate gastrointestinal comfort before exercise. The same foods and quantities relative to body mass were provided before each fed-condition session, and meal consumption was supervised by the research team. No additional caloric food or beverage was permitted between completion of the meal and testing. Final water intake was scheduled approximately 60 min before testing, after which no further food or fluid was consumed. Participants were instructed to avoid caffeinated beverages, energy drinks, and nutritional supplements for at least 24 h and to refrain from vigorous exercise, structured training, and competitive activity for 48 h before every testing session.

### 2.5 Testing procedures

All participants completed the same testing procedures under both fed and Ramadan-type fasted conditions. Testing was organized into two separate sessions for each condition. The first session included short-duration field-based performance tests, whereas the second session included the intermittent endurance test. This structure was used to prevent residual fatigue from the jump, sprint, and change-of-direction tests from influencing Yo-Yo test performance.

At the beginning of each Yo-Yo testing session, participants completed a brief health check and reported their fasting duration, time since the last meal, and time since the last fluid intake. Participants and their parents were instructed to maintain the child's usual sleep schedule and provide an opportunity for at least 8 h of nocturnal sleep before testing. Bedtime, wake time, and total sleep duration for the preceding night were recorded in a standardized sleep diary completed with parental assistance. Sleep duration was calculated as the interval between reported sleep onset and final awakening. Resting heart rate and pre-test subjective fatigue were also recorded only on the Yo-Yo testing day in each condition. Before each testing session, participants completed a standardized 10-min warm-up supervised by the same investigator. It consisted of 5 min of low-intensity running, approximately 2 min of dynamic lower-limb mobility exercises, and three progressive 20-m runs performed at approximately 60%, 75%, and 90% of perceived maximal running speed. Before countermovement-jump testing, participants additionally performed two submaximal practice jumps. A 3-min passive recovery period was provided between completion of the warm-up and the first maximal test. Before the Yo-Yo session, participants completed the same general warm-up without the countermovement-jump practice trials. The identical warm-up procedure was used in both nutritional conditions.

In the intermittent endurance session, participants performed the Yo-Yo Intermittent Recovery Children's Test on a separate testing day. The same pre-test procedures were applied before the Yo-Yo test. Immediately after completion of the Yo-Yo test, perceived exertion was recorded using a 0–10 scale. All tests were performed on the same type of surface and under similar testing conditions whenever possible.

### 2.6 Measurements

Body height, body mass, and body composition were assessed during the familiarization visit to characterize the baseline anthropometric profile of the participants. Body height was measured using a portable stadiometer and recorded to the nearest 0.1 cm. Body mass and body-fat percentage were assessed using a Tanita MC-780MA bioelectrical impedance analyzer (Tanita Corp., Tokyo, Japan), and body mass index was calculated as body mass divided by height squared and expressed as kg/m^2^. In addition to these baseline anthropometric measurements, body mass was measured separately for the condition comparison immediately before the warm-up on the Yo-Yo testing day in both the fed and Ramadan-type fasted conditions, at approximately 17:00. Participants were barefoot and wore the same type of light sports clothing at each assessment. Body mass was recorded to the nearest 0.1 kg. The same device and standardized procedure were used in both conditions.

Resting heart rate was recorded on the Yo-Yo testing day using a Huawei Watch GT5 (Huawei Technologies Co., Ltd., Shenzhen, China). Participants remained seated quietly for 5 min in a shaded area and were instructed to remain silent and avoid unnecessary movement. Resting heart rate was recorded as the stabilized value obtained at the end of the 5-min seated rest period. The same device was used for each participant in both conditions. Because measurement was obtained using a consumer wrist-worn device rather than electrocardiography, resting-heart-rate findings were interpreted as supportive contextual information rather than a definitive measure of cardiovascular strain.

Before the Yo-Yo test, participants rated their current subjective fatigue using a verbally administered 0–10 numerical rating scale, where 0 represented ‘not tired at all’ and 10 represented ‘extremely tired.’ Immediately before scoring, the investigator explained the anchors using age-appropriate language and confirmed that each child understood the scale. This scale was used as a simple contextual measure of perceived fatigue rather than as a validated diagnostic instrument. Immediately after the Yo-Yo test, perceived exertion was recorded using a child-appropriate 0–10 perceived-exertion scale, where 0 represented ‘very easy’ and 10 represented ‘extremely hard.’ The same standardized verbal instructions and scale anchors were used in both conditions. Child-specific perceived-exertion scales have demonstrated acceptable validity during locomotor exercise in children of comparable age (24, 25).

Countermovement jump performance was assessed using the flight-time function of the My Jump Lab mobile application (release 2.0) installed on an Apple iPhone 12 Pro running iOS 15.1. Video recordings were obtained at 240 frames/s. The smartphone was mounted on a tripod approximately 3.0 m from the participant and 0.75 m above ground level, with the camera positioned perpendicular to the sagittal plane. The field of view provided uninterrupted visualization of the feet during take-off and landing. Take-off was identified as the first frame in which both feet completely lost contact with the ground, and landing as the first frame in which ground contact was re-established. Flight time was determined by manual frame selection within the application, from which jump height was calculated. Participants began from an upright standing position, performed a rapid self-selected countermovement, and jumped vertically as high as possible. Two maximal trials were performed with 60 s of passive recovery, and the highest jump was retained for analysis (26, 27).

Linear sprint performance was assessed using a 30-m maximal sprint test. Sprint time was measured using an electronic photocell timing-gate system (Witty, Microgate Srl, Bolzano, Italy). Timing gates were positioned at the start and 30-m finish lines, with the photocell beams set approximately 1.0 m above ground level. Participants adopted a stationary split-stance starting position 0.5 m behind the first timing gate to prevent premature triggering. They were instructed to accelerate maximally and continue sprinting at maximal effort through the finish line. Two maximal trials were performed with 3 min of passive recovery, and the fastest time was retained for analysis. The same timing-gate placement and testing configuration were used in all sessions.

Change-of-direction performance was assessed using the standard Illinois Agility Test. The course was established within a 10 × 5 m area, with four central cones positioned 3.3 m apart along the longitudinal axis in accordance with the standard Illinois configuration. Completion time was measured using the same electronic photocell timing-gate system (Witty, Microgate Srl, Bolzano, Italy). The timing gates were positioned at the start/finish line with the photocell beam approximately 1.0 m above ground level. Participants completed the prescribed course as rapidly as possible. Two maximal trials were performed with 5 min of passive recovery, and the fastest time was retained for analysis. Course dimensions, cone positions, surface, and timing-gate configuration were identical across sessions (28, 29).

Intermittent endurance performance was assessed using the Yo-Yo Intermittent Recovery Level 1 Children's Test on the same artificial-turf surface with a standardized audio-paced protocol. Participants completed repeated 2 × 20-m shuttle runs at progressively increasing speeds, with a 10-s active recovery comprising a 2 × 2.5-m recovery shuttle between successive running bouts. Running speed was controlled by standardized audio signals. The test was terminated when a participant twice failed to reach the designated line within the required time or voluntarily stopped because of exhaustion. Total distance completed before termination was recorded in meters. The protocol has demonstrated high test-retest reliability in football-trained children of comparable age (21, 30).

### 2.7 Control variables and safety procedures

Condition-specific fasting duration, time since the last meal, and time since the last fluid intake were recorded on the Yo-Yo testing day in each condition to support interpretation of the intermittent-endurance outcome. Sleep duration, resting heart rate, and pre-test subjective fatigue were likewise collected on the Yo-Yo testing day only.

All testing sessions were conducted on the same artificial-turf soccer field under comparable environmental conditions. Ambient temperature and relative humidity were measured immediately before each session using a portable digital thermo-hygrometer positioned adjacent to the testing area. Ambient temperature ranged from 20 to 23 degrees C. Testing was conducted at approximately the same time of day (17:00 +/- 30 min) in both conditions. A minimum of 72 h and a maximum of 96 h separated consecutive experimental sessions. Participants completed no structured soccer training, competitive matches, or vigorous exercise during the 48 h preceding each session.

Participant safety was monitored throughout all testing procedures. Before each session, a brief health check was performed, and testing was postponed or discontinued if a participant reported illness, injury, dizziness, unusual fatigue, or any discomfort that could increase exercise-related risk. During testing, participants were allowed to stop at any time. The test was immediately terminated if a participant reported dizziness, nausea, excessive fatigue, discomfort, or unwillingness to continue. Research staff were present throughout the sessions to supervise the procedures and ensure participant safety.

### 2.8 Statistical analysis

Data were analyzed using the Simplified Statistics Program (KIP; version 1.0) (31). Descriptive data are presented as mean ± standard deviation. Normality was assessed for within-participant difference scores using the Shapiro–Wilk test. Normally distributed paired outcomes were compared using two-tailed paired-samples t-tests. Pre-test subjective fatigue, post-Yo-Yo perceived exertion, and sleep duration were analyzed using two-tailed Wilcoxon signed-rank tests because their paired difference scores did not meet the normality assumption. Paired mean differences were calculated consistently as fasted minus fed. For normally distributed outcomes, 95% confidence intervals were calculated using the t distribution; for non-normally distributed outcomes, bias-corrected and accelerated bootstrap 95% confidence intervals based on 200,000 resamples were reported. Statistical significance was accepted at *p* < 0.05. Yo-Yo Intermittent Recovery Children's Test distance was prespecified as the primary outcome; all remaining variables were secondary outcomes. No multiplicity adjustment was applied to the prespecified primary outcome. Secondary findings were interpreted with emphasis on effect estimates and confidence intervals rather than solely on statistical significance. Effect sizes were calculated as the bias-corrected standardized paired difference, Hedges' g, using J(df) x [mean paired difference/standard deviation of paired differences], with the same fasted-minus-fed sign convention throughout (32).

## 3 Results

Descriptive statistics and comparisons between fed and Ramadan-type fasted conditions are presented in Table 1. No statistically significant differences were detected between conditions for countermovement jump height, 30-m sprint time, or Illinois agility test time.

**Table 1:** Differences in performance and perceptual responses between fed and fasted conditions.

Variables	Fed	Fasted	Test statistic	*p*	Hedges' g	Paired mean difference (95% CI)
Countermovement jump height (cm)	24.8 ± 3.2	24.7 ± 3.1	t = -0.604	0.556	-0.15	-0.08 (-0.36 to 0.20)
30-m sprint time (s)	5.55 ± 0.32	5.58 ± 0.32	t = 1.501	0.156	0.37	0.03 (-0.01 to 0.08)
Illinois agility test time (s)	19.66 ± 0.95	19.81 ± 0.97	t = 1.507	0.154	0.37	0.16 (-0.07 to 0.38)
Yo-Yo Intermittent Recovery Children's Test distance (m)	610.7 ± 123.7	581.3 ± 113.8	t = -5.047	< 0.001	-1.23	-29.33 (-41.80 to -16.87)
Resting heart rate (bpm)	82 ± 5	84 ± 5	t = 6.883	< 0.001	1.68	2.07 (1.42 to 2.71)
Pre-test subjective fatigue (0–10 AU)	1.9 ± 0.9	2.7 ± 1.00	W = 0.000	0.002	1.28	0.87 (0.53 to 1.20)
Post-Yo-Yo perceived exertion (0–10 AU)	7.07 ± 1.16	8.4 ± 0.74	W = 0.000	0.001	1.74	1.33 (0.93 to 1.67)
Sleep duration (h)	8.4 ± 0.5	7.8 ± 0.8	W = 3.000	0.002	-1.38	-0.63 (-0.82 to -0.39)
Body mass (kg)	36.9 ± 3.7	36.2 ± 4.2	t = -3.241	0.006	-0.79	-0.63 (-1.04 to -0.21)

Values are presented as mean ± SD. RPE, rating of perceived exertion; AU, arbitrary units. Paired mean differences and 95% CIs were calculated as fasted minus fed. Test statistics are t for paired-samples t-tests and W for Wilcoxon signed-rank tests. Bias-corrected and accelerated bootstrap 95% CIs are reported for pre-test subjective fatigue, post-Yo-Yo perceived exertion, and sleep duration; t-distribution-based 95% CIs are reported for the remaining outcomes. Hedges' g was calculated as J(df) × [mean of the paired differences/SD of the paired differences] and follows the same sign convention. Sleep duration, resting heart rate, and pre-test subjective fatigue represent measurements collected on the Yo-Yo testing day in each condition.

Yo-Yo Intermittent Recovery Children's Test distance was significantly lower under the Ramadan-type fasted condition than under the fed condition (Table 1).

Resting heart rate, pre-test subjective fatigue, and post-Yo-Yo perceived exertion were significantly higher under the Ramadan-type fasted condition than under the fed condition (Table 1).

Sleep duration and body mass were significantly lower under the Ramadan-type fasted condition than under the fed condition (Table 1). Ambient temperature was comparable between conditions (fed: 21.6 +/- 1.4 degrees C; fasted: 21.7 +/- 1.5 degrees C). In the fasted condition, mean food-abstinence duration was 14.2 +/- 0.4 h and mean time since the last fluid intake was 13.9 +/- 0.4 h. In the fed condition, the mean interval between completion of the standardized meal and testing was 3.7 +/- 0.2 h, and the final water intake occurred 1.0 +/- 0.1 h before testing.

Figure 1 presents the paired individual observations for the four field-based performance outcomes. The individual trajectories show comparatively small and variable within-participant changes in countermovement jump, 30-m sprint, and Illinois agility performance, whereas Yo-Yo distance generally decreased under the Ramadan-type fasted condition.

**Figure 1:**
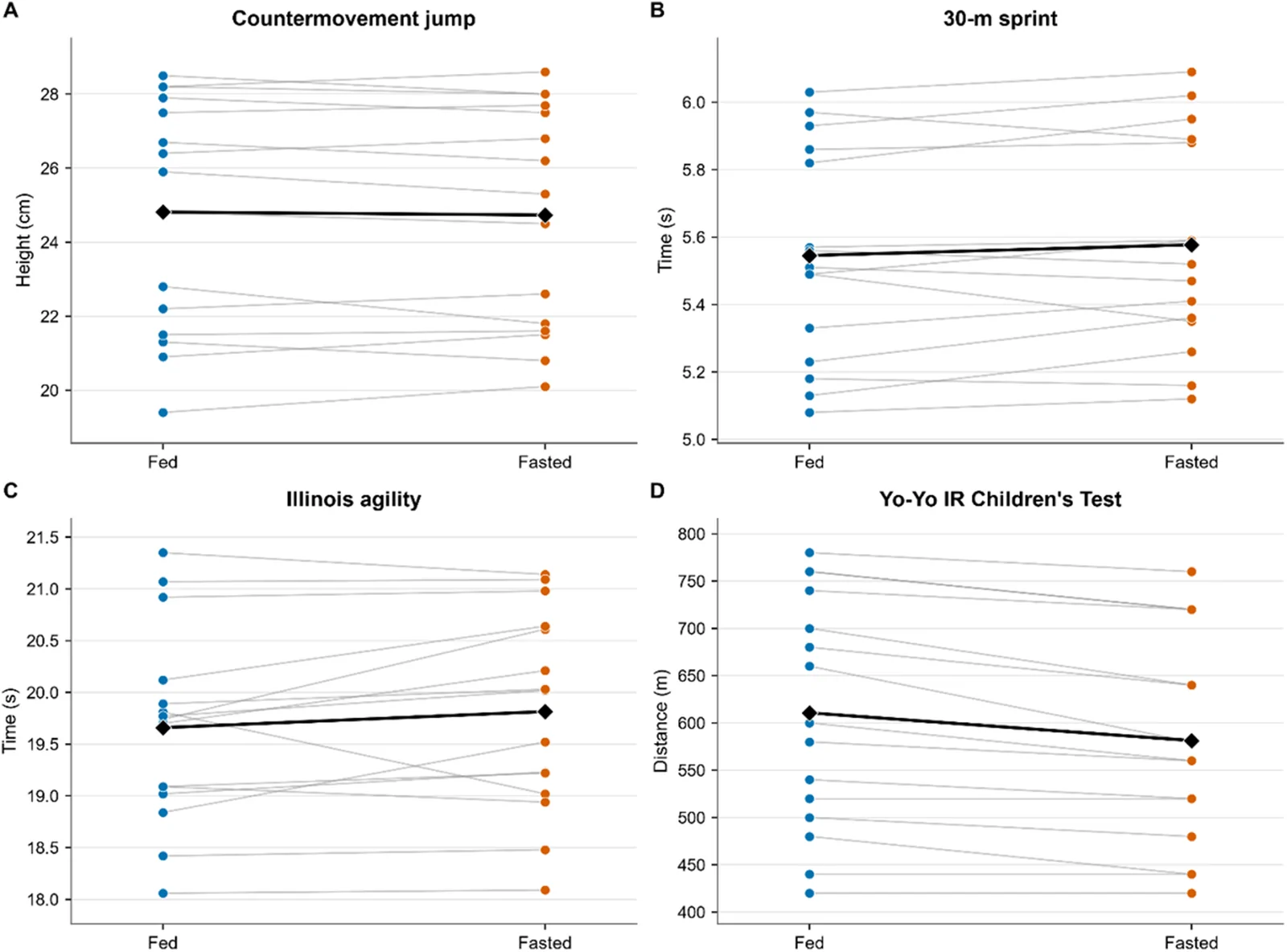
Paired individual performance responses under fed and ramadan-type fasted conditions. Panels show **(A)** countermovement jump height, **(B)** 30-m sprint time, **(C)** Illinois agility test time, and **(D)** Yo-Yo Intermittent Recovery Children's Test distance. Each grey line connects the fed and fasted observations for one participant (*n* = 15). Blue and orange circles represent individual fed and fasted values, respectively; black diamonds and the connecting black line represent condition means. Individual paired observations are displayed to illustrate the direction and variability of within-participant responses.

## 4 Discussion

The present study compared field-based physical performance, physiological and perceptual responses, sleep duration, and body mass between fed and Ramadan-type fasted conditions in 10–11-year-old male youth soccer players. The main findings were that countermovement jump height, 30-m sprint time, and Illinois agility test time did not differ significantly between conditions, whereas Yo-Yo Intermittent Recovery Children's Test distance was significantly lower under the Ramadan-type fasted condition. In addition, resting heart rate, pre-test subjective fatigue, and post-Yo-Yo perceived exertion were significantly higher in the fasted condition, while sleep duration and body mass were significantly lower. Overall, these findings suggest that the Ramadan-type fasted condition was associated with domain-specific responses, with the most evident performance difference observed in intermittent endurance rather than isolated short-duration performance tasks.

No statistically significant differences were detected in countermovement jump, 30-m sprint, or Illinois agility performance between conditions. However, these findings should not be interpreted as evidence that short-duration performance was unaffected or maintained, because the sample provides limited precision for detecting small-to-moderate differences. This finding is consistent with previous reviews reporting that Ramadan fasting does not consistently impair short-term maximal performance outcomes in soccer players, including vertical jump, sprint, maximal voluntary contraction, handgrip strength, and agility performance (33). Similarly, meta-analytic evidence suggests that jump height and strength-related outcomes are generally less affected by Ramadan fasting than repeated sprint or longer-duration exercise tasks (1). A plausible explanation is that these tests are brief, single-effort or near-single-effort tasks that rely largely on neuromuscular coordination, rapid force production, and immediate energy supply. Accordingly, no statistically significant decrement in countermovement jump, sprint, or change-of-direction performance was detected in the present testing context following approximately 14 h of food and fluid abstinence. However, this interpretation should be limited to the current testing context, as fasting-related responses may vary with age, training status, testing time, sleep duration, hydration status, and the cumulative demands of the exercise protocol.

The significant reduction in Yo-Yo Intermittent Recovery Children's Test distance under the Ramadan-type fasted condition represented the main performance difference observed in the present study. This finding is consistent with previous evidence in football players showing that Ramadan intermittent fasting may be associated with decrements in high-intensity or intermittent running performance, particularly when testing is performed in the late afternoon or evening before food and fluid intake is resumed (34). Unlike single-effort tasks such as jumping or sprinting, Yo-Yo performance requires repeated shuttle running, brief recovery periods, sustained pacing, and tolerance to accumulating physiological and perceptual strain. A possible explanation is that prolonged food abstinence may have reduced carbohydrate availability, while repeated high-intensity shuttle running progressively increased reliance on endogenous carbohydrate stores. Lower blood glucose or reduced glycogen availability could impair the ability to sustain repeated efforts and recover during the brief intervals. Because neither blood glucose nor muscle glycogen was measured, this explanation remains hypothetical; developmental differences in substrate metabolism should also be considered in children (10, 11, 35). Therefore, the lower Yo-Yo distance observed under the fasted condition may reflect the greater sensitivity of intermittent endurance performance to the combined demands of repeated high-intensity running and prolonged food and fluid abstinence. This interpretation is also supported by recent work showing that Ramadan-type fasting can increase internal physiological and perceptual load during matched high-intensity intermittent soccer exercise, even when external workload and neuromuscular performance are maintained (8). In this context, the present findings suggest that intermittent endurance performance may be more affected than isolated short-duration performance tasks under Ramadan-type fasting in child soccer players.

The higher resting heart rate, pre-test subjective fatigue, and post-Yo-Yo perceived exertion observed under the Ramadan-type fasted condition provide additional context for interpreting the reduction in intermittent endurance performance. These findings support greater perceived strain under the Ramadan-type fasted condition; however, evidence for greater physiological strain is limited because heart rate during the Yo-Yo test was not recorded and the available cardiovascular measure was restricted to resting heart rate. This interpretation is consistent with recent evidence showing that Ramadan-type fasting can increase internal physiological and perceptual load during matched high-intensity intermittent soccer exercise, as reflected by higher heart rate responses and session rating of perceived exertion despite comparable external workload (8). In the present study, the higher post-Yo-Yo perceived exertion may indicate that the endurance-based task was experienced as more demanding under the fasted condition, which may have contributed to the lower tolerance for repeated high-intensity running. However, this explanation should remain cautious because direct physiological mechanisms were not assessed. In addition, resting heart rate was measured using a wrist-worn monitor rather than an electrocardiography-based or chest-strap system; therefore, this physiological finding should be interpreted as a supportive contextual measure rather than a definitive marker of cardiovascular strain.

The lower sleep duration observed under the Ramadan-type fasted condition provides an important contextual factor for interpreting the higher subjective fatigue and post-Yo-Yo perceived exertion. Previous studies in athletic populations have shown that Ramadan fasting can alter sleep-wake patterns, including reduced sleep duration, delayed sleep onset, poorer sleep quality, increased daytime sleepiness, and greater insomnia symptoms (3, 36). Similar findings have also been reported in adolescent athletes, where Ramadan fasting worsened sleep-related indices while short-duration physical fitness tests remained relatively stable (37). Therefore, although sleep was not the primary outcome of the present study, the lower sleep duration in the fasted condition may have contributed to the higher perceived fatigue and greater exertional strain during the Yo-Yo test. Body mass was also lower under the fasted condition, which is consistent with previous evidence indicating that body mass and body composition-related indices may decrease during Ramadan fasting in athletes (2). However, because direct hydration markers such as urine specific gravity, urine osmolality, plasma osmolality, or blood-based hydration markers were not assessed, the lower body mass observed under the fasted condition should not be interpreted as direct evidence of dehydration.

From a practical perspective, the present findings suggest that coaches and practitioners should carefully plan and monitor intermittent endurance-oriented activities in child soccer players who voluntarily perform Ramadan-type fasting with parental approval. In the present study, the paired observations showed small and variable changes in the short-duration outcomes, whereas Yo-Yo distance decreased by an average of 29.3 m (approximately 4.8%) under the Ramadan-type fasted condition. The practical importance of this difference should be interpreted against the test-retest reliability, typical error, or minimal detectable change reported for the specific children's Yo-Yo protocol: Castagna et al. (2019) reported excellent relative reliability (ICC = 0.94) and a typical error of 5.1% for the Yo-Yo Intermittent Recovery Level 1 Children's Test (30), whereas Póvoas et al. (2016) reported a typical error of 11.1% (95% CI: 9.0–14.7%) in 9- to 11-year-old football players (21). Because the observed 4.8% mean reduction was smaller than both reported typical-error estimates, its practical magnitude should be interpreted cautiously, despite the statistically significant paired comparison. This pattern is consistent with evidence in football players showing that performance decrements during Ramadan fasting are more commonly observed during late afternoon or evening testing, before food and fluid intake is resumed, and during high-intensity exercise (34). Therefore, when children are fasted, demanding intermittent running sessions may require closer monitoring of perceived fatigue, perceived exertion, sleep duration, and recovery status. Previous reviews also suggest that maintaining energy and macronutrient intake, training load, body composition, sleep duration, and sleep quality may reduce the likelihood of substantial performance decrements during Ramadan fasting (38). In addition, recovery-oriented strategies, such as optimizing sleep opportunities, may be useful, as a short nap opportunity has been shown to improve physical and cognitive performance during Ramadan-related testing contexts (39). Importantly, these recommendations apply only to children who already fast voluntarily with parental approval and should not be interpreted as encouragement for children to fast for sport, training, or research purposes.

The present study has several strengths and limitations that should be considered when interpreting the findings. A major strength is the within-subject design, in which the same participants completed both fed and Ramadan-type fasted conditions. This design reduced the potential influence of inter-individual variability and allowed a more direct comparison of performance responses between conditions. Another strength is that testing was conducted at a similar time of day across conditions, helping to minimize the potential influence of diurnal variation. In addition, short-duration performance tests were separated from the Yo-Yo Intermittent Recovery Children's Test to reduce the possible effect of residual fatigue on intermittent endurance performance. The study also focused on a specific and practically relevant age group of youth soccer players, for whom field-based evidence regarding Ramadan-type fasting remains limited.

Several limitations should be considered. First, the final sample comprised only 15 male soccer players aged 10–11 years and did not reach the sample size required by the revised two-tailed power calculation, limiting statistical precision and generalizability. Second, the fasted condition represented an acute Ramadan-type fasting model rather than repeated fasting across the full month of Ramadan. Third, sleep duration was significantly shorter under the fasted condition; therefore, the conditions differed not only in food and fluid availability but also in sleep, and the observed differences cannot be attributed exclusively to fasting. Fourth, hydration status was not assessed using direct markers such as urine specific gravity, urine osmolality, or plasma osmolality; consequently, lower body mass under the fasted condition should not be interpreted as direct evidence of dehydration. Fifth, exercise heart rate was not recorded during the Yo-Yo test, and resting heart rate was measured using a consumer wrist-worn device. Sixth, biological maturity was not assessed, and maturation-related differences in metabolism and physical performance could not be accounted for. Finally, although the within-subject design reduced inter-individual variability, analyses were restricted to children who completed both nutritional conditions, and selection bias related to willingness to complete the fasting protocol cannot be excluded.

## 5 Conclusion

The present study detected no statistically significant differences in countermovement jump, 30-m sprint, or Illinois agility performance between fed and Ramadan-type fasted conditions in 10–11-year-old male youth soccer players. However, intermittent endurance performance, assessed by the Yo-Yo Intermittent Recovery Children's Test, was significantly lower under the fasted condition. The fasted condition was also accompanied by higher resting heart rate, greater pre-test subjective fatigue, higher post-Yo-Yo perceived exertion, lower sleep duration, and lower body mass. These findings indicate that the Ramadan-type fasted condition was associated with lower intermittent endurance performance and greater perceptual strain, whereas no statistically significant differences were detected in isolated short-duration performance tasks in child soccer players. Coaches and practitioners should therefore carefully monitor fatigue, perceived exertion, sleep, and recovery when planning intermittent endurance-oriented activities for children who voluntarily fast with parental approval.

## Data Availability

The datasets generated and analyzed during the current study are available from the corresponding author upon reasonable request.
